# Proteomic Analysis of *Fasciola hepatica* Excretory and Secretory Products Co-Immunoprecipitated Using Time Course Infection Sera

**DOI:** 10.3390/pathogens10060749

**Published:** 2021-06-13

**Authors:** Zhuo Lan, Xiao-Lei Liu, Qing-Bo Lv, Min-Hao Zeng, Jun-Feng Gao, Qiao-Cheng Chang, Yuan-Yuan Chen, Chun-Ren Wang

**Affiliations:** 1 College of Animal Science and Veterinary Medicine, Heilongjiang Bayi Agricultural University, Daqing 163319, China; zhuolan@byau.edu.cn (Z.L.); qingbolv@byau.edu.cn (Q.-B.L.); zengminhao@stu.just.edu.cn (M.-H.Z.); gaojunfeng@byau.edu.cn (J.-F.G.); qcchang@stu.edu.cn (Q.-C.C.); chunrenwang@byau.edu.cn (C.-R.W.); 2 Heilongjiang Provincial Technology Innovation Center for Bovine Disease Control and Prevention, Daqing 163319, China; 3 Key Laboratory of Zoonosis Research, Ministry of Education, Institute of Zoonosis, College of Veterinary Medicine, Jilin University, Changchun 130012, China; liuxlei@jlu.edu.cn

**Keywords:** *Fasciola hepatica*, *Fasciola gigantica*, excretory and secretory products, Co-IP

## Abstract

*Fasciola hepatica* is a widespread pathogen that is known for its harmful effects on the health and productivity of ruminant animals. To identify the proteins present in all periods of infection with *F. hepatica* but not in those with *Fasciola gigantica* by shotgun liquid chromatography–tandem mass spectrometry (LC–MS/MS), we collected the ESPs and sera of *F. hepatica* and *F. gigantica*. In this study, the sheep were artificially infected with *F. hepatica* and the sera were collected at five different periods: 3 days post-infection (dpi), 7 dpi, 21 dpi, 63 dpi, and 112 dpi. The interacting proteins were pulled down from the sheep sera of all five periods and the sera with *F. gigantica* by co-immunoprecipitation (Co-IP) assay, before being identified by LC–MS/MS analysis. Thirty, twenty-two, twenty-three, twenty-seven, and twenty-two proteins were pulled down by the infected sera at 3 dpi, 7 dpi, 21 dpi, 63 dpi, and 112 dpi, respectively. Among them, 12 proteins existed in all periods, while six proteins could be detected in all periods in *F. hepatica* but not in *F. gigantica*. Protein relative pathway analysis revealed that these proteins mainly refer to the metabolism, regulation of genetic activity, and signal transduction of *F. hepatica*. In conclusion, this study provides meaningful data for the diagnosis of fasciolosis and to understand the interactions between *F. hepatica* and the host.

## 1. Introduction

*Fasciola hepatica* mainly parasitizes the liver and bile ducts of ruminant animals and humans, causing fasciolosis. Fasciolosis is widely seen in Europe, Asia, America, and Africa and is also observed in other areas of the world [1]. It is estimated to cause annual economic losses >USD 3.2 billion dollars worldwide [2]. *F. hepatica* can cause acute and chronic hepatitis and cholangitis in animals, accompanied by systemic poisoning and nutritional disorders. When cattle and sheep are infected with *F. hepatica*, there is a decrease in the quality of their biological products and milk yield, which poses a remarkable threat to animal husbandry [3]. Humans are also at risk of fasciolosis, with approximately 2.6 million infected people globally [4]. Helminths are powerful immunoregulators. Excretory and secretory products (ESPs) are a mixture of proteins, lipids and carbohydrates which are secreted and excreted by the parasites (either soluble secreted proteins or those packaged within extracellular vesicles) and those expressed on the outer surface of the tegument during parasitism of the host [5]. Antigenic stimulation is an important initiating factor for the generation, maintenance, and regulation of host immune responses. As worms from different sources have their own specific antigen components, the mechanisms of action and host immune responses in them are not identical [6]. Antigens are directly exposed to the host immune system. ESPs are not only one of the main antigens that stimulate humoral and cell-mediated immunity, but they also play important roles in the survival of parasites and host–parasite interactions [7]. Compared to the antigens, ESPs of flukes have better sensitivity and specificity, and the antibody levels in hosts are positively correlated with the severity of infection caused by the flukes [8,9]. Proteomics and bioinformatics can be used to analyze the dynamic changes in excretory and secretory proteins expressed in different periods of infection, which can provide information for the screening and identification of important antigen molecules which may be related to the induction of host immune responses, immune regulation, immune escape, etc. Using proteomics approaches based on mass spectrometry, the protein composition of ESPs has been characterized in several species, including *Haemonchus contortus*, *Heligmosomoides polygyrus*, *Ascaris suum*, and *Nippostrongylus brasiliensis* [10,11,12]. Scholars have studied the changes of protein components in small cell lung cancer H446 cells treated with ESPs of *Trichinella spiralis* by LC–MS/MS, and showed that the anti-tumor mechanism of ESPs and their processes are complex [13]. Gourbal and Jefferies performed proteomic analysis of the *F. hepatica* excretory and secretory products (FhESPs), and Jefferies identified 29 proteins, including cathepsin L and proteins related to immune escape. Global data on the components, proportion, and relative abundance of FhESPs have also been analyzed [14,15]. Moreover, some learners take proteomic analysis of *Fasciola gigantica* ESPs interacting with the buffalo serum of different infection periods by shotgun LC–MS/MS, and they identified some proteins which provides material for studies about the interaction between *Fasciola gigantica* and host [16]. However, given the difficulty in obtaining parasites at different developmental stages and the fact that these parasites can excrete or secrete different antigens during different stages of their development in the host, information regarding FhESPs is still very limited. Fortunately, we can address these limitations owing to a recent report on the *Fasciola* genome and associated transcriptome datasets [17]. In addition, there is the other *Fasciola* genome which improved annotation for the trematode *Fasciola hepatica* [18]. We can also perform a definitive characterization of the total antigenic targets of adult *F. hepatica* in different infection periods.

The purpose of this study was to identify the antigenic targets in FhESPs using an immunoproteomic approach. The immunoprecipitants were analyzed and characterized using liquid chromatography–tandem mass spectrometry (LC–MS/MS). This approach can be used to analyze specific proteins and provide a reliable basis for the screening of diagnostic antigens of *F. hepatica*.

## 2. Materials and Methods

### 2.1. FhESPs Preparation

FhESPs were prepared according to the standard procedure previously described by Novobilsky et al. [19]. Adults of *F. hepatica* were separated from the liver and washed three times in phosphate-buffered saline (PBS) to remove the host material. The flukes were then incubated in sterile RMPI 1640 medium with the addition of antibiotics and antimycotic (10,000 UI/mL penicillin G, 10 mg/mL amphotericin B) at 37 °C for 2 h. *F. hepatica* was then transferred into a new medium with the same RMPI 1640 and incubated for 23 h at 37 °C. The supernatant was obtained at 5 h, 11 h and 23 h centrifuged (13,400× *g* for 20 min), they were combined then stored at −80 °C until further use.

### 2.2. Preparation of Serum

Six sheep were selected from Daqing, Heilongjiang Province, China. All animal studies were performed in accordance with the Guide for the Care and Use of Laboratory Animals (1996). This study was approved by the Animal Health, Animal Care and Use Committee of Heilongjiang BAYI Agricultural University. Ivermectin and albendazole were orally administered for one month. *F. hepatica* metacercariae were obtained from the eggs from naturally infected sheep in Heilongjiang, China. Wet lettuce leaves with 220 metacercaria were wrapped into a mass to keep them moist and then randomly fed to three sheep with a mouth opener. The sheep were also given some water to ensure that they swallowed the metacercaria along with the lettuce leaves. After 60 days of infection, the feces of infected sheep were collected for fecal examination. At 3, 7, 21, 63, and 112 dpi, blood samples were aseptically collected from animal into tubes without anticoagulants. The sera were separated by centrifugation and preserved at −80 °C for further use. We selected the positive serum of different periods from all the positive sera of three infected sheep. We obtained the negative serum from the other three sheep at 21 days, 63 days and 112 days, which were uninfected. The *F. gigantica* serum at 21 dpi, 63 dpi and 112 dpi of three sheep were obtained from the Laboratory Animal Center of Lanzhou Veterinary Research Institute, Chinese Academy of Agriculture Sciences. The *F. gigantica* serum of each sheep at different stages were pooled separately. Target proteins that interacted with the positive serum of sheep at different infection stages and *F. gigantica* serum of sheep were pulled down by co-immunoprecipitation (Co-IP).

### 2.3. Co-IP of FhESPs-Antibody Binding Proteins

The Protein A/G PLUS Agarose immunoprecipitation kit (Santa Cruz Biotechnology, USA) was used for Co-IP according to the manufacturer’s instructions. Additionally, the Co-IP was performed in biological triplicate. The sheep normal serum and the serum at 3 dpi, 7 dpi, 21 dpi, 63 dpi, and 112 dpi, as well as the *F. gigantica* serum, were placed on ice and diluted with 200 μL PBS. The 30 μL protein A/G plus agarose beads were washed three times with 200 μL PBS buffer, divided into 21 tubes, added to the samples and incubated at 4 °C for 1 h and centrifuged at 1000× *g* for 30 s. Thereafter, the supernatant was carefully discarded and the beads were washed three times with 200 μL PBS buffer. The FhESPs were filtered and concentrated, and the immunoprecipitation (IP) lysate was added for cleavage and quantification. Subsequently, 500 μg FhESPs was added to the protein A/G beads along with 500 μL PBS. The beads were then incubated at 4 °C for 1.5 h and centrifuged at 1000× *g* for 30 s. The supernatant was discarded and the beads were washed three times with 200 μL PBS buffer. Thereafter, 20 μL 5 × SDS-loading was added and the mixture was centrifuged at 95 ℃ for 10 min, and then centrifuged at 1000× *g* for 30 s to collect the supernatant. Fifteen micrograms of each sample was analyzed by sodium dodecyl sulfate–polyacrylamide gel electrophoresis (SDS-PAGE).

### 2.4. In-Gel Trypsin Digestion

The gel in each lane was cut into pieces of 1 mm^3^ with a scalpel, placed into different 1.5 mL tubes, and washed with 200 μL distilled water twice, for 10 min each time. The dye decolorizing solution (50 mm NH_4_HCO_3_ and ACN 1:1) was then added for 15 min and washed with double distilled water. This was repeated three times until the decolorization was complete. A 100 μL volume of acrylonitrile (ACN) was added to dehydrate the solution until the colloidal particles turned white and vacuum was added for 10 min to make it dry. Thereafter, 200 μL of 10 mM dithiothreitol (DTT) (25 mM NH_4_HCO_3_ dissolved) was added and placed in a water bath at 37 °C for 1 h. Then, 100 μL ACN was added to dehydrate the solution until the colloidal particles turned white and 200 μL of 55 mM IAA (25 mM NH_4_HCO_3_ dissolved) was added. It was placed in the dark room for 30 min. Then, 100 μL ACN was added to dehydrate the solution until the colloidal particles turned white and the suspension was successively washed with double distilled water, ACN for 10 min for each liquor, finally washed with double distilled water for 10 min again. Then, 100 μL of 0.01 μg/μL trypsin working solution (the enzyme solution was diluted with 25 mM NH_4_HCO_3_) was added to each tube and slightly centrifuged. The sample was freeze-dried into a powder.

### 2.5. LC–MS/MS Analysis

The lyophilized samples were dissolved in 20 μL of 2% methanol and 0.1% formic acid (FA) by centrifugation at 1000× *g* for 20 min and the supernatant was collected. Mass spectrometry (MS) data were acquired with Q Exactive (ThermoFinnigan, San Jose, CA, USA). Briefly, loading peptides onto a reverse phase trap column (Thermo Scientific Acclaim PepMap100, 100 μm × 2 cm, nano Viper C18) that was pre-equilibrated with 0.1% FA. An analytical column (Thermo Scientific Easy Column, 12 cm long, 75 μm inner diameter, 3 μm resin) was used to separate components with a linear gradient of buffer B (100% *v/v* acetonitrile and 0.1% *v/v* formic acid) at a flow rate of 3 μL per min for 8 min. The separation flow rate was 600 nL per min.

### 2.6. Data Analysis

Specific proteins were analyzed according to the Proteome Discoverer 2.4.1.15. The raw file of the mass spectrum was identified and analyzed using the commercial software, MaxQuant (Thermo Fisher Scientific, Waltham, MA, USA). The search parameters were as follows: value of the enzyme was trypsin, static modification was C-carboxyamidomethylation (57.021 Da), dynamic modification was oxidation (M), species was *F. hepatica*, mass tolerance of the precursor ion was ±15 ppm, fragment ion mass tolerance was ±0.5 Da, and the protein false discovery rate (FDR) was set at 0.01. The maximum number of missed cleavages was 2. The Kyoto Encyclopedia of Genes and Genomes (KEGG) analysis of the specific proteins of *F. hepatica* was performed to select the most significant pathway and to analyze the relationship between the abundance of specific proteins of different pathways and the different periods of infection.

## 3. Results and Discussion

**Figure 1:**
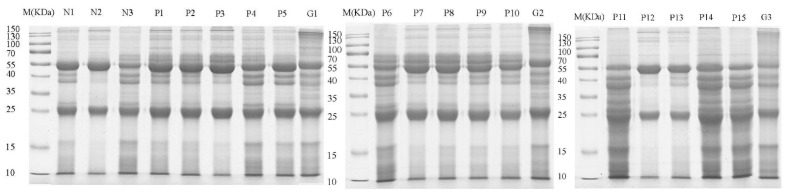
The sodium dodecyl sulfate–polyacrylamide gel electrophoresis (SDS-PAGE) analysis of samples with the sheep serum from different infection periods co-cultured with the *F. hepatica* excretory and secretory products (FhESPs) and the serum with *F. gigantica*. Line N1~N3: proteins pulled down by three negative sheep serum with *F. hepatica*. Line P1~P5: proteins pulled down by the first infected sheep serum of 3 days post-infection (dpi), 7 dpi, 21 dpi, 63 dpi and 112 dpi with *F. hepatica*. Line G1: proteins pulled down by the first *F. gigantica* serum. Line P6~P10: proteins pulled down by the second sheep serum of 3 dpi, 7 dpi, 21 dpi, 63 dpi and 112 dpi with *F. hepatica*. Line G2: proteins pulled down by the second *F. gigantica* serum. Line P11~P15: proteins pulled down by the third sheep serum of 3 dpi, 7 dpi, 21 dpi, 63 dpi and 112 dpi with *F. hepatica*. Line G3: proteins pulled down by the third *F. gigantica* serum.

**Figure A1:**
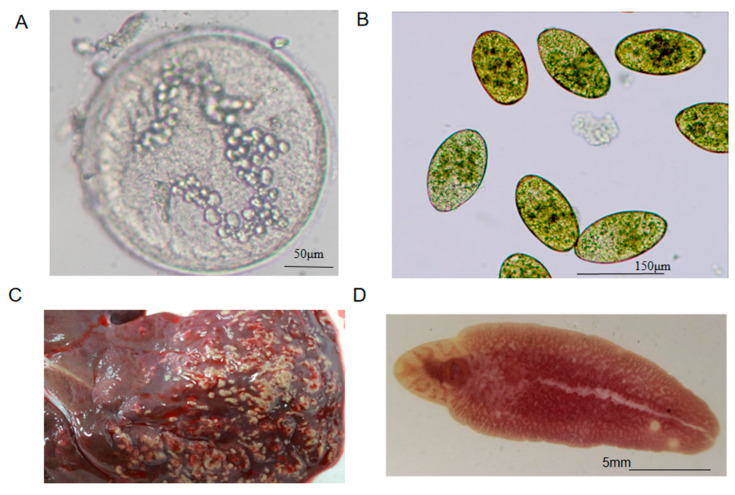
Infection characteristics: (**A**) Metacercariae used to infect sheep; (**B**) *F. hepatica* eggs observed under an optical microscope; (**C**) the liver of sheep infected with *F. hepatica*; (**D**) the *F. hepatica* collected from the liver of sheep previously infected with *F. hepatica*.

There are cases of the human infection, fascioliasis, in 56 countries, mainly in Bolivia, Peru, Egypt, Portugal and China [20]. Fascioliasis not only seriously affects the development of animal husbandry, but also poses a threat to human health. Therefore, the prevention and control of fascioliasis are of utmost importance. There is a clear need and global interest in the development of improved methods for controlling fascioliasis. We collected the *F. hepatica* metacercariae from the *Galba pervia* (Figure A1A). The results of the fecal examination along with the morphology of the eggs are shown in Figure A1B. There were pathological changes in the liver and various nodules could be seen in (Figure A1C). *F. hepatica* was collected (Figure A1D). Sheep infected with *F. hepatica* were successfully established. The albendazle pretreatment was taken to remove other helminths and that only *Fasciola hepatica* eggs were found following facial examination. The serum will be specific which guarantees the accuracy of the data we obtained. An immunoproteomic approach was used to identify the proteins secreted by *F. hepatica*, specifically using infection serum to pull down the FhESPs that are likely to be involved in host–parasite interactions. SDS-PAGE was used to confirm the results of the Co-IP assay (Figure 1). The results indicated that the antibodies from the serum of different infection periods could recognize and pull down specific proteins from FhESPs. The figures about proteins which are available seem very low given the SDS-PAGE because *F. gigantica* sera and negative serum were removed from the analysis. The proteins we expected to obtain need to exist in *F. hepatica* but not in *F. gigantica* sera, and are specific as compared with the negative serum. The majority of proteins had molecular weights ranging from 10 to 170 kDa.

**Figure 2:**
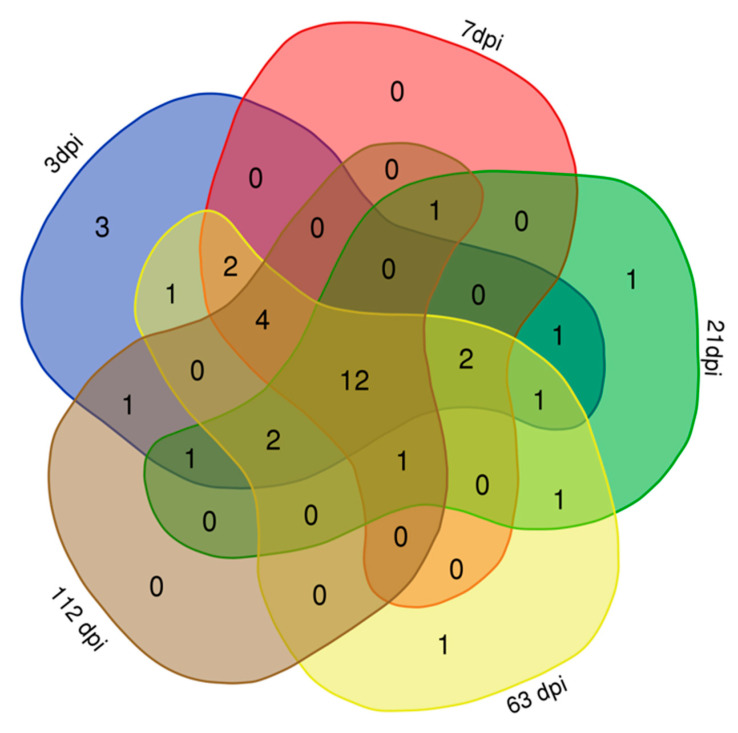
Total proteins that were identified to be binding to the sheep serum at: 3 days post-infection (dpi) with *F. hepatica* (blue); 7 dpi with *F. hepatica* (pink); 21 dpi with *F. hepatica* (green); 63 dpi with *F. hepatica* (yellow); and 112 dpi with *F. hepatica* (grey).

**Figure 3:**
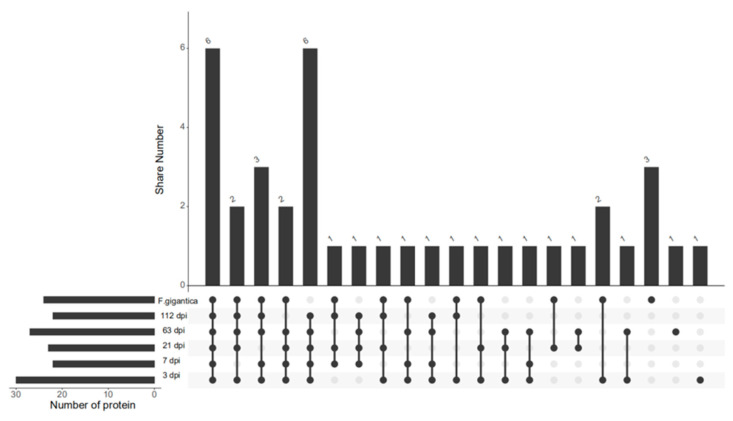
Total proteins that were identified to be binding to the sheep serum at 3 days post-infection (dpi); 7 dpi; 21 dpi; 63 dpi; and 112 dpi with *F. hepatica* and the total proteins that were identified to be binding to the sheep serum with *F. gigantica*.

**Figure 4:**
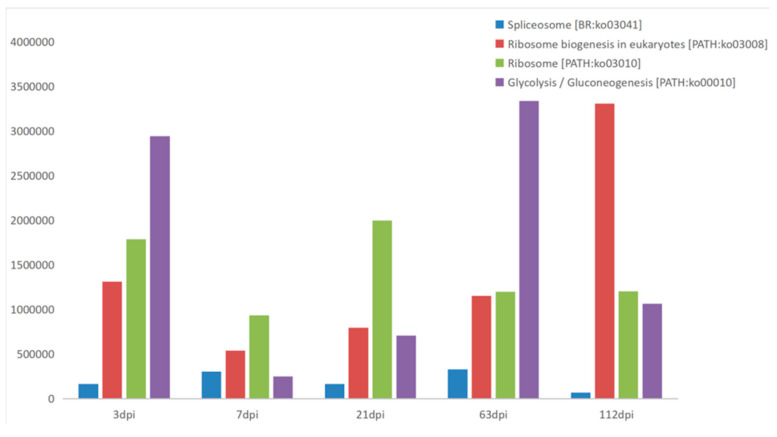
The selected signal path in the Kyoto Encyclopedia of Genes and Genomes (KEGG) database is displayed with different colors. Different periods are displayed by the horizontal coordinates. The abundance expressed by specific proteins is displayed by the longitudinal coordinates.

Comparing all results, we found that there were 30, 22, 23, 27, and 22 proteins identified at 3 dpi, 7 dpi, 21 dpi, 63 dpi, and 112 dpi, respectively, according to the LC–MS/MS analysis, but only 12 proteins were co-purified in all five periods (Figure 2, Table 1). The abundance of proteins are all well (Table A1). Some of these proteins are currently not annotated. However, some of the annotated proteins, such as acyl-coenzyme A thioesterase 8, are related to metabolism, while others may have different functions. In addition, upon comparing these protein datasets for different infection periods with those of *F. gigantica*, we found that there was an overlap in the protein expression levels among these samples, and the relationships of these pulled down proteins was summarized in an upsetvenn diagram (Figure 3). This indicated that six proteins might take part in the host–parasite interactions during the whole infection period, and they have good specificity compared to the proteins of *F. gigantica*. These six proteins are the last six proteins in Table 1. Two proteins were unannotated. The alpha subunit of casein kinase II is related to ATP-binding and belongs to the protein kinase superfamily. The casein kinase 2 alpha subunit (CK2a) is involved in the activation of muscle-specific gene programs. The CK2 subunits exert specific and coordinated functions in skeletal muscle differentiation and show fusogenic activity [21]. HIV Tat-specific factor 1 protein, which belongs to the HTATSF1 family, is related to RNA binding. Tat-SF1 is not required for regulating HIV-1 transcription, however, it is required to maintain the ratios of different classes of HIV-1 transcripts [22]. The fructose-bisphosphatase protein belongs to the fructose-1,6-bisphosphatase (FBPase) class 1 family, which is related to the fructose 1,6-bisphosphate-1-phosphatase activity (Table 1). The proteins of the Anisakis proteomes were characterized by label-free quantification and functional analysis, and proteins involved in many essential biological mechanisms, such as parasite survival, were identified, among which is the fructose 1,6-bisphosphatase [23]. The 40S ribosomal protein, S3a, is a structural constituent of the ribosome, which belongs to the eukaryotic ribosomal protein eS1 family. These proteins were detected in *F. hepatica* but not in *F. gigantica*, where they took part in the host–parasite interactions. The Kyoto Encyclopedia of Genes and Genomes (KEGG) is a reference knowledge base for the biological interpretation of large-scale molecular datasets [24]. KEGG revealed that the specific proteins were mainly involved in the metabolism and regulation of genetic activity and the proteins related to signal transduction and metabolism play important roles in the interaction between *F. hepatica* and the host. We also found that the abundance of proteins was different in each period of infection (Figure 4). The 40S ribosomal protein, S3a, which is related to the ribosomal pathway, was relatively higher and more stable than other proteins. CK2a was highest at 112 dpi in the ribosome biogenesis pathway and the fructose-bisphosphatase protein was highest at 64 dpi in the glycolysis/gluconeogenesis pathway in eukaryotes. Thus, specific proteins could be good candidates for further diagnostic studies of infections. Proteins with high expression, including the 40S ribosomal protein S3a, CK2a, and the fructose-bisphosphatase protein may be better for further diagnostic studies.

ESPs play important roles during the development of parasites, especially in host–parasite interactions, and many ESPs of *Fasciola spp.* have been shown to evade and defend against the host immune responses [15]. FhESPs can downmodulate the proliferation of spleen mononuclear cells [25] and induce immunomodulatory effects on macrophages by inducing T-cell activity via the selective upregulation of programmed death-ligand 2 (PD-L2) expression in a Dectin-1 dependent manner [26]. ESPs from *F. hepatica* are directly exposed to the host immune system and are widely used as antigens in serological assays. MM3-ELISA uses monoclonal antibodies to capture protein fragments (mainly cathepsin L1 and L2) of molecular weights 7–40 kDa from FhESPs. The sensitivity and specificity of this kit are 99% and 100%, respectively [6,27]. However, the cost performance of this kit was unsatisfactory. The proteins have been purified as part of complexes by Co-IP. Although the mixture of antibody and antigen is extracted, the antibody complex is incubated with the antigen derived from *F. hepatica,* then the *F. hepatica* can be identified in the search library by MS. However, whether the identified proteins screened in this study have high specificity and sensibility still needs to be proven by subsequent experiments including Western blot and ELISA. If they have good reaction in the following study, they have good antigenicity. Once some of them can be purified and have good combination with positive serum, then these can provide a good foundation for developing new immunological diagnostic methods. Many proteins were identified in this study, however, the functions of most of them are unknown. Moreover, the key genes involved in the interplay between the host and parasite interactions have not yet been identified. This highlights the central role played by ESPs in the protection of *F. hepatica* from the host immune responses. Therefore, this study lays a foundation for further studies on the interactions between *F. hepatica* and the host as well as the diagnosis of *F. hepatica*.

## Figures and Tables

**Table 1:** The *Fasciola hepatica* excretory and secretory products (FhESPs) which were detected in the sheep post-infection in all periods.

Protein Description	Species	Genome Mapping Gene ID ^a^	Peptides	Unique Peptides	Cover Percent	GeneBank Bank ID	MW ^b^(KDa)	calc. pI ^c^
Uncharacterized protein 1	*Fasciola hepatica*	D915_008782	2	2	12	THD20526.1	25.9	6.24
Heparosan-N-sulfate-glucuronate 5-epimerase	*Fasciola hepatica*	D915_004542	1	1	1	THD24727.1	80	9.03
Synaptophysin	*Fasciola hepatica*	D915_006334	1	1	3	THD22918.1	26.1	5.34
Uncharacterized protein 2	*Fasciola hepatica*	D915_001733	1	1	4	THD27516.1	15.5	7.03
Acyl-coenzyme A thioesterase 8	*Fasciola hepatica*	D915_007699	1	1	2	THD21305.1	37.1	6.87
ATP-dependent Clp protease ATP-binding subunit clpX mitochondrial	*Fasciola hepatica*	D915_009354	1	1	1	THD19712.1	60.5	6.35
Uncharacterized protein 3	*Fasciola hepatica*	D915_000294	1	1	2	THD28860.1	30.9	6.99
Uncharacterized protein 4	*Fasciola hepatica*	D915_005102	1	1	1	THD24171.1	55.6	8.76
40S ribosomal protein S3a	*Fasciola hepatica*	D915_000892	1	1	3	THD28280.1	25.3	9.85
HIV Tat-specific factor 1 protein	*Fasciola hepatica*	D915_000726	1	1	1	THD28435.1	67.5	5
Fructose-bisphosphatase	*Fasciola hepatica*	D915_007520	1	1	5	THD21694.1	29.1	6.16
Alpha subunit of casein kinase II	*Fasciola hepatica*	D915_004801	1	1	2	THD24445.1	44.2	8.48

^a^ The genome mapping gene ID only relates to *Fasciola hepatica.* ^b^ Molecular weight of the leading protein sequence contained in the protein group. ^c^ Theoretical isoelectric point.

## Data Availability

The data presented in this study are available on request from the corresponding author.

## Appendix B

**Table A1:** The proteins derived from each period and their abundance.

GenBank	Protein Description	Abundance				
		3 dpi	7 dpi	21 dpi	63 dpi	112 dpi
THD21074.1	Gelsolin repeat protein	24,022.21289	—	—	—	—
THD22536.1	Fructose-bisphosphate aldolase	86,937.625	—	172,559.1563	—	—
THD27725.1	Uncharacterized protein	553,797.5	—	793,536.625	—	504,179.2188
THD23308.1	Dynein light chain	11,747.43164	14,691.50586	—	7394.90625	21,221.78906
THD24816.1	Serpin protein	1,646,911.188	—	134,091.1719	333,078.8438	906,792.8125
THD27667.1	Phosphoglucomutase	341,188.4844	—	—	417,641.75	—
THD20135.1	Legumain	2,782,378	—	—	—	17,424,414.5
THD20526.1	Uncharacterized protein	77,763.89844	53,412.98438	79,610.59375	44,011.75781	116,365.3672
THD22651.1	Glucose-6-phosphate 1-dehydrogenase	161,910.9531	—	170,645.4531	536,310.6875	—
THD26293.1	Annexin	488,116.9375	—	—	—	—
THD21694.1	Fructose-bisphosphatase	2,940,358.5	250,116.7344	706,553.3125	3,337,365.5	1,063,860
THD23211.1	Tubulin alpha chain	330,362.1563	—	130,189.7266	768,367.25	243,900.8594
THD28435.1	HIV Tat-specific factor 1 protein	163,905.5781	302,068.5313	164,833.7969	328,743.75	67,329.42188
THD24727.1	Heparosan-N-sulfate-glucuronate 5-epimerase	409,968.5	85,834.78125	160,129.6875	155,940	156,170.7813
THD22918.1	Synaptophysin	119,084.1016	68,156.35938	103,948.9063	222,138.8828	87,811.85156
THD28852.1	Ubiquitin-conjugating enzyme	668,924.625	118,031.6016	NA	505,793.9375	343,043.6172
THD27516.1	Uncharacterized protein	4,501,626	926,071.1875	2,201,982.75	1,899,086.625	1,882,076.625
THD24445.1	Alpha subunit of casein kinase II	1,313,028.719	537,602.3906	793,033.75	1,153,075.125	3,306,170.313
THD19712.1	ATP-dependent Clp protease ATP-binding subunit clpX mitochondrial	3,510,484.25	—	—	—	—
THD22278.1	Tubulin--tyrosine ligase-like protein 9	473,730.9688	—	—	—	—
THD28860.1	Uncharacterized protein	49,954,940	14,922,535	18,942,136	40,814,652	38,499,836
THD26528.1	rRNA-processing protein FCF1 isogeny	263,285.7188	110,388.4141	—	325,662.4688	—
THD25229.1	Hexosyltransferase	100,760.8984	29,143.81445	—	26,410.00195	—
THD22369.1	Uncharacterized protein	188,394.8125	62,918.48828	—	93,628.02344	101,414.2734
THD24171.1	Uncharacterized protein	499,705.5625	93,921.17969	293,650.7813	1,253,055.875	694,243.1875
THD21345.1	Uncharacterized protein	389,435	503,797.4063	2,321,797.25	602,599.5625	—
THD21879.1	Inhibitor of apoptosis protein	1,977,777.75	1,209,314.875	1,346,054	1,589,138.125	—
THD28280.1	40S ribosomal protein S3a	1,786,953.672	934,144.1875	1,994,597.219	1,198,627.375	1,202,573
THD21305.1	Acyl-coenzyme A thioesterase 8	1,959,986.75	162,016.5469	185,430.125	1,942,750.313	1051,204.156
THD28550.1	Uncharacterized protein	335,545.5625	39,628.06641	—	181,182.0156	118,531.6094
THD28078.1	Guanine nucleotide exchange factor	—	44,768.47266	466,165.8438	—	236,903.5234
THD19712.1	ATP-dependent Clp protease ATP-binding subunit clpX mitochondrial	—	221,215.0469	985,376.8125	2,183,246.25	2,075,125.5
THD20617.1	ATP-dependent DNA helicase	—	417,392.3438	442,980.4375	286,577.8125	481,421.625
THD19133.1	VIT domain-containing protein	—	—	93,653.66406	51,209.55859	—
THD19784.1	ML domain-containing protein	—	—	65,019.27344	—	—
THD28903.1	Isocitrate dehydrogenase [NADP]	—	—	—	770,771.625	—
